# Improved resilience following digital cognitive behavioral therapy for insomnia protects against insomnia and depression one year later

**DOI:** 10.1017/S0033291722000472

**Published:** 2022-03-08

**Authors:** Philip Cheng, David A. Kalmbach, Hsing-Fang Hsieh, Andrea Cuamatzi Castelan, Chaewon Sagong, Christopher L. Drake

**Affiliations:** 1 Thomas Roth Sleep Disorders and Research Center, Henry Ford Health System, 39450 W 12 Mile Road, Novi, MI 48197, USA; 2 Department of Health Behavior and Health Education, School of Public Health, University of Michigan, 1415 Washington Heights, Ann Arbor, MI 48109 USA

**Keywords:** CBT-I, depression, insomnia, mobile health, resilience

## Abstract

**Background.:**

While the negative consequences of insomnia are well-documented, a strengths-based understanding of how sleep can increase health promotion is still emerging and much-needed. Correlational evidence has connected sleep and insomnia to resilience; however, this relationship has not yet been experimentally tested. This study examined resilience as a mediator of treatment outcomes in a randomized clinical trial with insomnia patients.

**Methods.:**

Participants were randomized to either digital cognitive behavioral therapy for insomnia (dCBT-I; *n* = 358) or sleep education control (*n* = 300), and assessed at pre-treatment, post-treatment, and 1-year follow-up. A structural equation modeling framework was utilized to test resilience as a mediator of insomnia and depression. Risk for insomnia and depression was also tested in the model, operationalized as a latent factor with sleep reactivity, stress, and rumination as indicators (aligned with the 3-P model). Sensitivity analyses tested the impact of change in resilience on the insomnia relapse and incident depression at 1-year follow-up.

**Results.:**

dCBT-I resulted in greater improvements in resilience compared to the sleep education control. Furthermore, improved resilience following dCBT-I lowered latent risk, which was further associated with reduced insomnia and depression at 1-year follow-up. Sensitivity analyses indicated that each point improvement in resilience following treatment reduced the odds of insomnia relapse and incident depression 1 year later by 76% and 65%, respectively.

**Conclusions.:**

Improved resilience is likely a contributing mechanism to treatment gains following insomnia therapy, which may then reduce longer-term risk for insomnia relapse and depression.

## Introduction

As the most prevalent and burdensome sleep disorder (Ohayon, 2002; Roth, 2007; Roth & Roehrs, 2003), insomnia is a significant public health concern. Chronic insomnia impairs daytime functioning, wreaks havoc on multiple domains of health, and increases the risk of medical morbidities more than fivefold compared to healthy sleepers (Kalmbach, Pillai, Arnedt, & Drake, 2016b; Taylor et al., 2007). Left untreated, insomnia is also a robust risk factor for mental illness (Pigeon, Bishop, & Krueger, 2017), including depression (OR 3.95), substance use disorders (OR 7.18) (Breslau, Roth, Rosenthal, & Andreski, 1996), and even suicide controlling for depression (OR 1.30) (Bernert, Turvey, Conwell, & Joiner, 2014).

Though the destruction of insomnia has been well-documented, the contrast of a strengths-based approach to understanding the role of sleep in health promotion is still emergent and much-needed (Buysse, 2014). Nascent evidence suggests that sleep promotes protective physiological functions such as clearing of harmful neuronal waste (e.g. *β*-amyloid) (Winer et al., 2020) or myelination of neurons (Bellesi, Bushey, Chini, Tononi, & Cirelli, 2016; Bellesi et al., 2018). In contrast to a deficit model, a strengths-based approach examines whether promotion of sleep health may interrupt and prevent a worsening health trajectory, or perhaps even alter the trajectory toward an ‘upward spiral’ that eventually returns to or surpasses premorbid levels of health and functioning.

In a strengths-based approach, resilience is of particular interest because it is among the critical components that determine health trajectories following stressors (Bonanno, 2005). Though there are various definitions of psychological resilience, a core component is the ability to respond and recover from stressors (i.e. the root of ‘resilience’ is ‘resile’, which means ‘to bounce or spring back’) (Carver, 1998; Tusaie & Dyer, 2004). Resilience has been linked to maintaining health, wellbeing, and even thriving despite facing stressors. Resilience is also of interest as a therapeutic target because it represents an opportunity for prevention and early intervention. Under the diathesis-stress model of psychopathology, stress in combination with predispositional risk factors precipitates psychopathology; however, individuals with higher levels of resilience may be more likely to respond adaptively and recover from stress. This then protects against adverse outcomes via reduced intensity and duration of symptoms, or even avoiding illness altogether.

There is growing evidence that sleep and resilience are interconnected. First, those who report greater psychological resilience also exhibit more robust sleep (e.g. greater sleep efficiency, fewer and shorter awakenings after sleep onset, less light sleep, and more deep sleep) (Brand et al., 2014a, 2014b, 2016). Similarly, those with poor sleep also report less resilience. Not only do good sleepers report greater resilience than those with insomnia, high levels of resilience protected individuals against the effects of stress on sleep quality (Palagini et al., 2018). This is consistent with patient reports of being more easily frustrated during episodes of insomnia, and with evidence of poor cognitive and emotional coping (Morin, Rodrigue, & Ivers, 2003; Pillai, Roth, Mullins, & Drake, 2014; Voss, Kolling, & Heidenreich, 2006). Greater resilience may lead to appraisal of daily life events as less stressful (Morin et al., 2003) and decreased occurrence or prolongation of stressful life events (Hall Brown, Akeeb, & Mellman, 2015; Healey et al., 1981; Luo, Zhang, & Pan, 2013).

Under the stress-diathesis model, a lack of protective resilience allows stress – and maladaptive appraisals of stress – to serve as a precipitant of psychopathology. This includes the ‘3-P’ model of insomnia (Spielman, Caruso, & Glovinsky, 1987), which posits that insomnia is triggered by a combination of predispositional [e.g. sleep reactivity (Drake, Friedman, Wright, & Roth, 2011), familial risk (Drake, Cheng, Almeida, & Roth, 2017)] and precipitating factors (e.g. life stressors), which are then maintained by some perpetuating factors [e.g. rumination and poor coping (Pillai et al., 2014)]. Once elicited, insomnia further potentiates risk for other forms of psychopathologies, including depression (Baglioni et al., 2011; Li, Wu, Gan, Qu, & Lu, 2016), anxiety (Dolsen, Asarnow, & Harvey, 2014), and other psychiatric and medical conditions (Dolsen et al., 2014; Ford & Kamerow, 1989; Taylor, Lichstein, & Durrence, 2003; Taylor et al., 2007). Conversely, bolstering resilience may confer protection against stressors and interrupt the development of psychopathology; however, extant studies examining the relationship between sleep and resilience have largely been observational and correlational, thus limiting causal and/or directional inferences.

There is emerging evidence to suggest that resilience can be bolstered by improving sleep, which then confers protection against additional psychopathologies. For example, several studies have shown that cognitive behavioral therapy for insomnia (CBT-I) – the recommended first-line treatment for insomnia (Qaseem, Kansagara, Forciea, Cooke, & Denberg, 2016; Riemann et al., 2017) – has positive effects for comorbid depression despite the fact that CBT-I does not directly target depression and typically has far fewer sessions compared to CBT for depression. More recent studies have shown that treatment of insomnia also prevents the incidence of depression (Batterham et al., 2017; Cheng et al., 2019). Furthermore, we recently demonstrated that individuals with insomnia who received digital CBT-I prior to the COVID-19 pandemic exhibited less stress and depression during the pandemic relative to a control group despite comparable disruptions due to the pandemic (Cheng, Casement, Kalmbach, Castelan, & Drake, 2020a). These findings provided further support that improving insomnia may bolster resilience against future stress. However, the direct impact of CBT-I on resilience has not been adequately tested, nor has resilience been tested as a mechanism for positive outcomes following CBT-I. Without this, we are not able to establish resilience as a modifiable factor that can be targeted via sleep intervention to protect against a trajectory of cascading psychopathology.

To expand our strengths-based understanding of sleep health, this study examined resilience in adults with insomnia before and after receiving digital CBT-I (dCBT-I). The central hypothesis was that those who received dCBT-I would report greater resilience compared to those in the control group, and that improved resilience would confer protection from insomnia and depression at 1-year follow-up by reducing risk factors associated with these conditions. We first hypothesized that those in the dCBT-I group would report greater improvements in psychological resilience compared to those in the sleep education control. We then improved resilience from dCBT-I would further mitigate the risk factors of insomnia and depression, which would in turn confer protection from insomnia and depression at 1-year follow-up.

## Methods

Data for this study were obtained from participants recruited from six hospitals, 38 medical centers, and subscribers of a major health insurance company in southeastern Michigan. Recruitment occurred between 2016 and 2017, and utilized Internet-based methods (see Cheng et al., 2018, 2019 for more details). Eligible participants met DSM-5 (American Psychiatric Association, 2013) diagnostic criteria for chronic insomnia disorder. Exclusion criteria included diagnosed sleep disorders other than insomnia (e.g. restless legs, narcolepsy) or untreated obstructive sleep apnea, and diagnosed bipolar disorder or seizure disorder. Because the SPREAD trial (NCT02988375) included a depression prevention aim, individuals with high depression chronicity (self-reported daily or near daily depressed mood and anhedonia) were excluded. Those who reported suicidality during screening were further assessed using the Columbia-Suicide Severity Rating Scale (C-SSRS) via telephone within 24 h by research staff certified in conducting the C-SSRS and referred to psychiatric or emergency services when appropriate.

### Study design

**Fig. 1.**
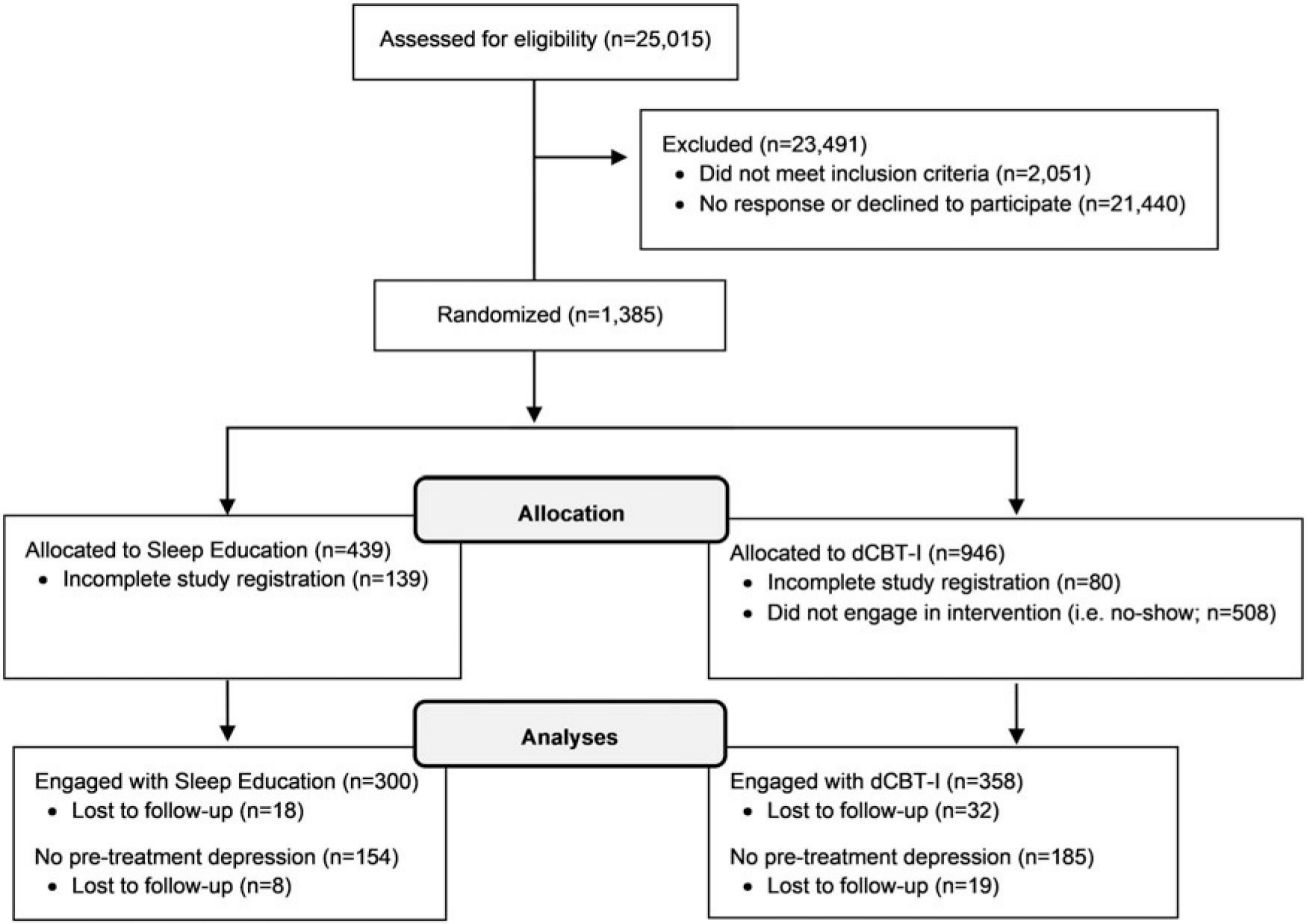
Enrollment flow chart.

This study utilized a randomized controlled design with simple randomization into two parallel arms: dCBT-I, or a control condition (online sleep education). Randomization was computerized and automated immediately after participants met eligibility criteria. A total of 1385 individuals with insomnia disorder were enrolled and randomized into either the dCBT-I or online sleep education conditions (see Fig. 1 for enrollment flow chart). The research staff was blinded to treatment allocation. Participants were randomized at a 2:1 ratio for the dCBT-I condition due to a higher anticipated attrition rate for the active compared to the sleep education condition, as has been previously demonstrated in nearly all Internet-based interventions (Christensen, Griffiths, & Farrer, 2009). The final sample included 358 participants in the dCBT-I treatment and 300 in the online sleep education condition (see Cheng, Kalmbach, Castelan, Murugan, & Drake, 2020b; Cheng et al., 2018 for additional details regarding study design and recruitment). All procedures were approved by the Institutional Review Board. Informed consent was also given by all participants immediately following study eligibility, before any study procedures were implemented.

### dCBT-I condition

Individuals randomized to the dCBT-I condition completed the Sleepio program via the Internet (www.sleepio.com, Big Health Ltd). Sleepio was selected among several programs for dCBT-I because it is evidence-based, fully automated, is standardized, and has been tested in almost 7000 participants via multiple RCTs (Barnes, Miller, & Bostock, 2017; Bostock, Luik, & Espie, 2016; Cheng et al., 2018; Espie et al., 2018, 2012; Freeman et al., 2017; McGrath et al., 2017; Pillai et al., 2015). Participants were provided each of the six core sessions of dCBT-I on a weekly basis, which were directed by an animated ‘virtual therapist’ who reviews and guides progress with the participant. Participants had access to the program for up to 12 weeks. The mean attendance rate was 4.1 sessions, and 43% completed all six sessions. The sessions predominantly targeted behavioral and cognitive components of insomnia (e.g. sleep restriction, stimulus control, cognitive restructuring, paradoxical intention), and included relaxation strategies (e.g. progressive muscle relaxation and autogenic training) and sleep hygiene in the first few sessions. The final session focused on relapse prevention.

### Online sleep education

Individuals randomized to the online sleep education condition received six weekly e-mails based on the NIH guide to healthy sleep (National Heart Lung and Blood Institute, 2011) containing information on the following topics: the basics of endogenous sleep regulation; the impact on sleep of health problems such as obesity, diabetes, and hypertension; the effects of sleep disruptive substances, such as caffeine, nicotine, alcohol; and tips on creating a sleep-conducive bedroom environment. Psychoeducation and sleep hygiene were selected because they are common in clinical practice, particularly in primary care (Buysse et al., 1997), and are commonly used as an attention control in clinical trials. Importantly, these are not considered effective standalone treatments for insomnia (Morgenthaler et al., 2006).

### Measures of interest

Resilience was measured using the Brief Resilience Scale (BRS) (Smith et al., 2008), which measures one’s ability to recover from stress. The BRS contains six items with responses on a fivepoint Likert scale. The items describe recovery in response to stress (e.g. ‘*It does not take me long to recover from a stressful event.*’), and responses range from Strong Disagree (1) to Strongly Agree (5). The BRS is scored using the mean of the six items with higher scores indicating greater psychological resilience. The BRS shows good test-retest reliability and construct validity (Smith et al., 2008). The BRS also showed strong internal reliability in this study (Cronbach’s *α* = 0.89). To support causal inferences in the mediation model via temporal precedence, psychological resilience as a mediator was operationalized as a change score from pre-treatment to post-treatment.

### Latent risk for insomnia and depression

In accordance with our interest in resilience as a transdiagnostic construct, we approach the latent risk factor also as a transdiagnostic construct. We were particularly interested in the protection that dCBT-I can confer beyond its well-established effects on symptom reduction. As such, we selected indicators risk based on a stress-diathesis model (in alignment with the 3-P model of insomnia) that also had prior evidence supporting a relationship with both insomnia and depression. Specifically, we examined how changes in resilience following dCBT-I impacted risk as measured by the confluence of transdiagnostic predisposing, precipitating, and perpetuating factors impacting both insomnia and depression. Under this model, all three factors (referred to as the ‘3-Ps’ in the 3-P model) are requisite and compound to increase the incidence of psychopathologies. Sleep reactivity (i.e. vulnerability to sleep disturbance) was selected as a predisposing factor as it has been demonstrated as a heritable predictor of both insomnia and depression (Drake et al., 2011; Drake, Pillai, & Roth, 2014; Harvey, Gehrman, & Espie, 2014; Kalmbach, Anderson, & Drake, 2018; Kalmbach, Pillai, Cheng, Arnedt, & Drake, 2015; Palagini et al., 2019). Stress was selected as a precipitating factor. Finally, a transdiagnostic measure of rumination was selected as a perpetuating factor (Ballesio, Ottaviani, & Lombardo, 2019; Carney, Harris, Moss, & Edinger, 2010; Malmberg & Larsen, 2015; Nolen-Hoeksema, 1991). These three variables were treated as indicators of a latent transdiagnostic risk factor that was then used as a predictor of insomnia and depression at 1-year follow-up.

Sleep reactivity describes a sleep system that is particularly vulnerable to perturbation. Sleep reactivity has been determined as a phenotype that shows stability over time (Jarrin, Chen, Ivers, Drake, & Morin, 2016) and is 30–40% heritable, suggesting a substantial genetic component (Drake et al., 2011). Sleep reactivity is measured using the Ford Insomnia Response to Stress Test (FIRST) (Drake, Jefferson, Roehrs, & Roth, 2006). The FIRST is a self-report measure of sleep reactivity that asks respondents to rate the likelihood (not, somewhat, moderately, and very likely) that they would experience sleep difficulties in reaction to nine hypothetical stressful situations (e.g. ‘*after a stressful experience during the day’, ‘before an important meeting the next day*’). Higher FIRST scores indicate a more highly sensitive sleep system. Participants with FIRST scores of 15 or below were characterized as having low sleep reactivity, and those who scored 16 or higher were characterized as having high sleep reactivity. The FIRST showed strong internal reliability (Cronbach’s *α* = 0.88).

Stress was measured using a validated single-item instrument (Elo, Leppänen, & Jahkola, 2003; Salminen, Kouvonen, Koskinen, Joensuu, & Väänänen, 2014). The prompt for this instrument was ‘*Stress means a situation in which a person feels tense, restless, nervous or anxious or is unable to sleep at night because his/her mind is troubled all the time. Do you feel this kind of stress these days?*’ Response on this instrument was on a five-point Likert scale ranging from never (0) to always (4), with higher scores indicating more stress.

Rumination was measured using the Perseverative Thinking Questionnaire (PTQ) (Ehring et al., 2011). The PTQ was selected as a transdiagnostic measure of rumination. The PTQ assesses how often individuals engage in repetitive negative thinking in response to negative experiences or problems using 15 items scored on a five-point Likert scale (0 = Never, 4 = Almost always). The PTQ has strong reliability and convergent validity with other validated rumination scales (e.g. Response Style Questionnaire, Rumination Scale), and with the measures of depression and anxiety (e.g. Beck Depression Inventory, Inventory of Depressive Symptomatology, State Trait Anxiety Inventory, Penn State Worry Questionnaire). As expected, the PTQ showed strong internal reliability in this study (Cronbach’s *α* = 0.96).

Insomnia severity was measured using the Insomnia Severity Index (ISI) (Morin, Belleville, Bélanger, & Ivers, 2011; Thorndike et al., 2011), with higher scores indicating increased insomnia severity (range 0–28). A score of 15 or higher is considered clinically significant insomnia. Items on the ISI in this study showed strong internal reliability (Cronbach’s *α* = 0.89). Depression was measured using the 16 item self-report Quick Inventory of Depressive Symptomatology (QIDS-SR_16_). This measure also showed strong internal reliability in this study (Cronbach’s *α* = 0.81). The QIDS-SR_16_ assesses the severity of the nine diagnostic symptom criteria used in the Diagnostic and Statistical Manual of Mental Disorders, and shows strong consistency with a diagnosis of major depressive disorder via a Structured Interview for the DSM (Lamoureux et al., 2010; Surís, Holder, Holliday, & Clem, 2016; Yeung et al., 2012). To examine changes in non-sleep symptoms of depression, analyses only utilized non-sleep items from the QIDS (items 1–4 were dropped) unless otherwise specified. Clinically significant depression was determined via a psychometrically derived threshold of 11 or higher on the total score across all items (corresponding to moderate severity or higher). Clinically significant depression was determined using a psychometrically derived threshold of 11 or higher (corresponding to moderate severity or higher). This cutoff has 82.4% sensitivity and 70.3% specificity with a SCID diagnosis of major depressive disorder (Lamoureux et al., 2010) and is consistent with clinical practices for screening and triaging patients for treatment or prevention.

### Analytical approach

This study aimed to elucidate treatment mechanisms, which is predicated on treatment engagement. Indeed, data from patients who never engage in treatment cannot elucidate the mechanisms of treatment. Therefore, we utilized an established intention-to-treat approach (Fergusson, Aaron, Guyatt, & Hébert, 2002; Gupta, 2011) that included individuals who engaged in at least one session of the allocated treatment. All individuals lost to follow-up were included in the analysis, and missing data were handled with full information maximum likelihood. This ITT approach was also adapted for circumstances where the risk for type II error is high. A critical weakness of the traditional ITT approach is its high susceptibility to type II error when attrition rate is high (Fergusson et al., 2002; Hollis & Campbell, 1999). Because non-usage attrition is an inherent and endemic problem in Internet-based treatments (Christensen et al., 2009; Melville, Casey, & Kavanagh, 2010; Neve, Collins, & Morgan, 2010) – with non-usage rates as high as 81% (Melville et al., 2010) – the traditional ITT approach would excessively increase type II error for this trial. The rationale for this adaptation is that data from those without any treatment exposure will indicate very little about the efficacy of the treatment (Gupta, 2011), and at worst generate erroneous parameter estimates leading to biased results.^†1^

**Fig. 2.**
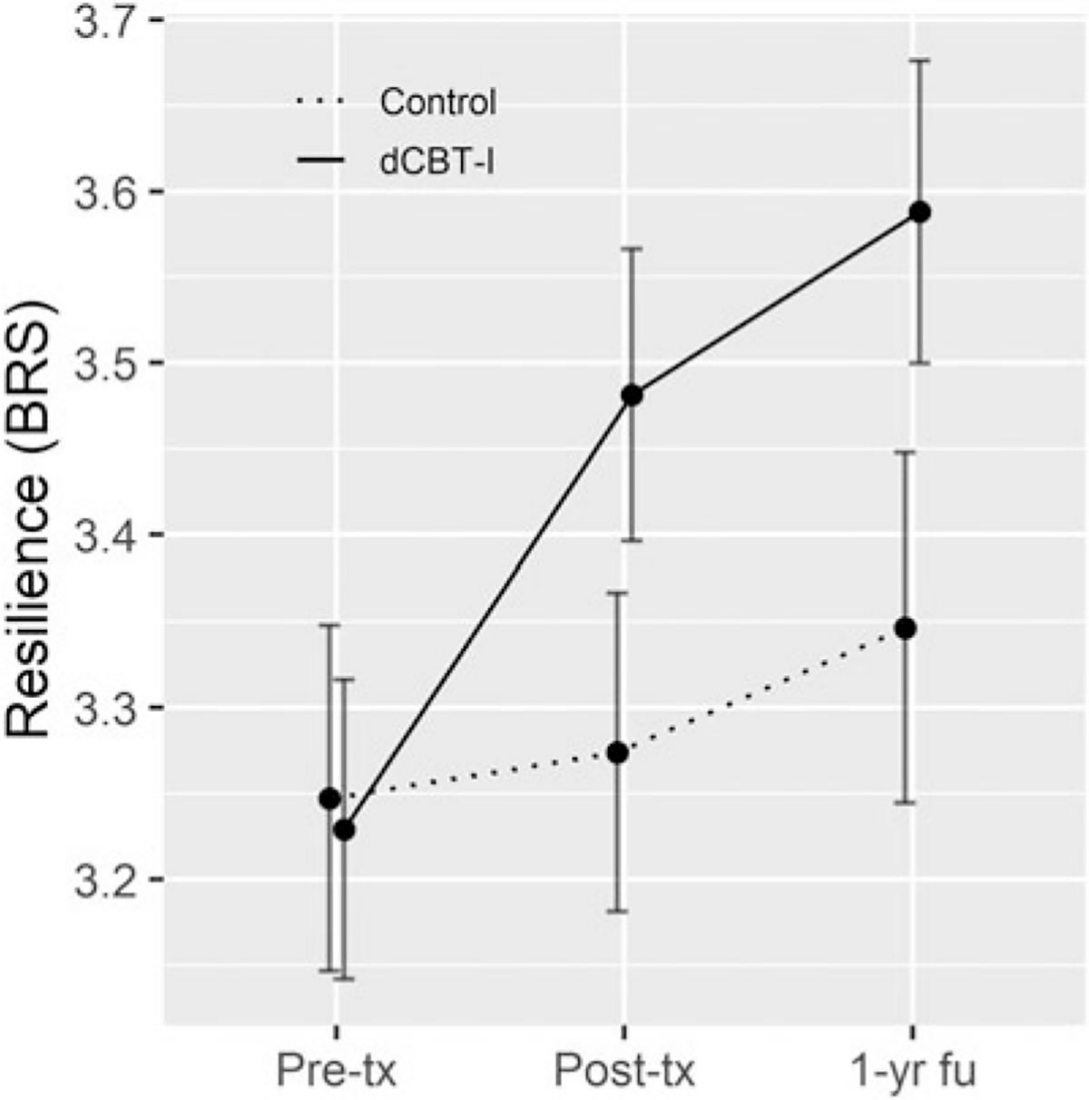
Resilience by group at each study timepoint. Pre-tx, pre-treatment; Post-tx, post-treatment; 1-yr fu, 1-year follow-up. Error bars indicate 95% confidence intervals. Cohen’s *d* of improved resilience from pre-tx due to dCBT-I: at post-tx = 0.34, at 1-year follow-up = 0.36.

**Fig. 3.**
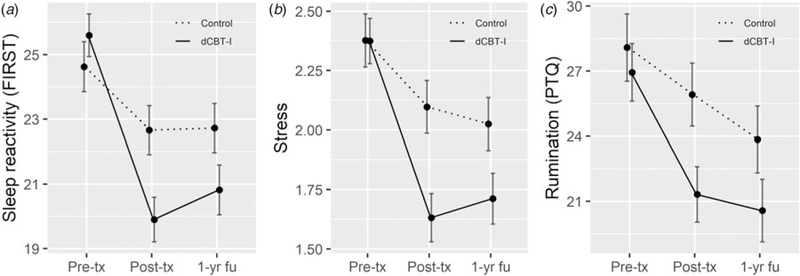
Means of sleep reactivity, stress, and rumination as risk factors of insomnia and depression over the three assessment timepoints (pre-treatment, post-treatment, and 1-year follow-up). dCBT-I, digital cognitive behavioral therapy for insomnia; FIRST, Ford Insomnia Response to Stress Test; PTQ, Perseverative Thinking Questionnaire; Pre-tx, pre-treatment; Post-tx, post-treatment; 1-yr fu, 1-year follow-up. Error bars represent 95% confidence intervals. Cohen’s *d* of improved risk indicators from baseline due to dCBT-I at post-treatment: *d*_FIRST_ = 0.55, *d*_stress_ = 0.44, *d*_rumination_ = 0.32; at 1-year follow-up: *d*_FIRST_ = 0.45, *d*_stress_ = 0.33, *d*_rumination_ = 0.21.

The first hypothesis tested the direct effect of dCBT-I on resilience via a mixed-effects linear regression with individuals as a random intercept to account for individual differences in baseline values. The second hypothesis tested the path models using structural equation modeling. Two sets of models were conducted separately by syndrome: one with insomnia as the outcome variable of interest (see Fig. 2) and the other with depression as the outcome variable of interest (see Fig. 3). Transdiagnostic risk for insomnia and depression was treated as a latent factor in the model with sleep reactivity, stress, and rumination as indicators of latent risk. The second hypothesis protection of resilience through reducing risk factors was tested via the indirect effect of dCBT-I on outcomes at 1-year follow-up through the impact of resilience on latent risk. Full information maximum likelihood was used to produce robust parameter estimates of the indirect effects, and statistical significance was tested using a 95% confidence interval of the unstandardized coefficients (indicated as *b* in the results) derived from bootstrapping drawing from 1000 samples. Standardized coefficients are also reported (indicated as *β* in the results). Model fit for each model was determined using four indices: the χ^2^ fit statistic, the root mean square error of approximation (RMSEA) <0.05 (Browne & Cudeck, 1992), the Tucker–Lewis index (TLI) >0.95, and the comparative fit index (CFI) >0.95 (Bentler, 1990; Tucker & Lewis, 1973). Pre-treatment scores were included during model building and were excluded from the final model for parsimony because it did not impact results. All analyses were conducted in R (version 4.0.5) using the LAVAAN package.

Sensitivity analyses were conducted in two subsamples to examine the effect of improved resilience on prevention more directly. The first sensitivity analysis examined the impact of resilience on insomnia relapse in a subsample that achieved remission at post-treatment (*n* = 364). Because resilience improved with insomnia remission, we opted to use change in resilience from post-treatment to 1-year follow-up to disentangle resilience from insomnia remission. The second sensitivity analysis similarly examined the impact of resilience on depression prevention in a subsample with minimal to no depression at baseline (*n* = 269).

## Results

At baseline, individuals in both conditions exhibited clinically significant insomnia of moderate severity. The sample also showed comorbid depression symptoms as is common in insomnia (see Table 1). Individuals reported moderate levels of sleep reactivity, stress, and rumination at baseline. As was previously reported (Cheng et al., 2018, 2020b), the dCBT-I condition resulted in acute reductions in both insomnia (10.0 ± 5.7 S.D. point decrease on the ISI) and depression symptoms (4.8 ± 5.0 S.D. point decrease on the QIDS-SR16) that were approximately twofold that in the control condition, with effects maintained at 1-year follow-up.

### dCBT-I improves resilience mediated through improved sleep

In support of the first hypothesis, results indicated that dCBT-I led to higher resilience at both post-treatment [*t*(1262.2) = 4.0, *p* < 0.001], and 1-year follow-up [*t*(1272.3) = 4.9, *p* < 0.001; see Fig. 2 for means and effect sizes across time points]. A post-hoc analysis confirmed that higher resilience following dCBT-I was mediated by improvements in insomnia symptom severity (indirect effect = 0.14, 95% CI 0.08–0.23; *β* = 0.17).

### Measurement model

Before testing our second hypothesis, we examined a measurement model to ensure the variables included were related to one another before computing the full structural model. A latent factor of risk for insomnia and depression was constructed with sleep reactivity, stress, and rumination as indicators. The factor model showed strong factor loadings for each of the three indicators (sleep reactivity = 0.70; stress = 0.77; rumination = 0.77; see Fig. 3 for means and effect sizes of the indicators across all time points). Other key constructs included change in resilience from pre- to post-treatment, depression, and insomnia outcomes. We allowed latent factors and key constructs to correlate with each other in the measurement model. The measurement model fit indices represent adequate model fit for insomnia [χ^2^ (8, *N* = 658) = 13.8, CFI = 0.99; TLI = 0.99; RMSEA = 0.03] and depression [χ^2^ (8, *N* = 658) = 12.1, CFI = 0.99; TLI = 0.99; RMSEA = 0.03].

### Improved resilience as a protective mechanism against insomnia

**Fig. 4.**
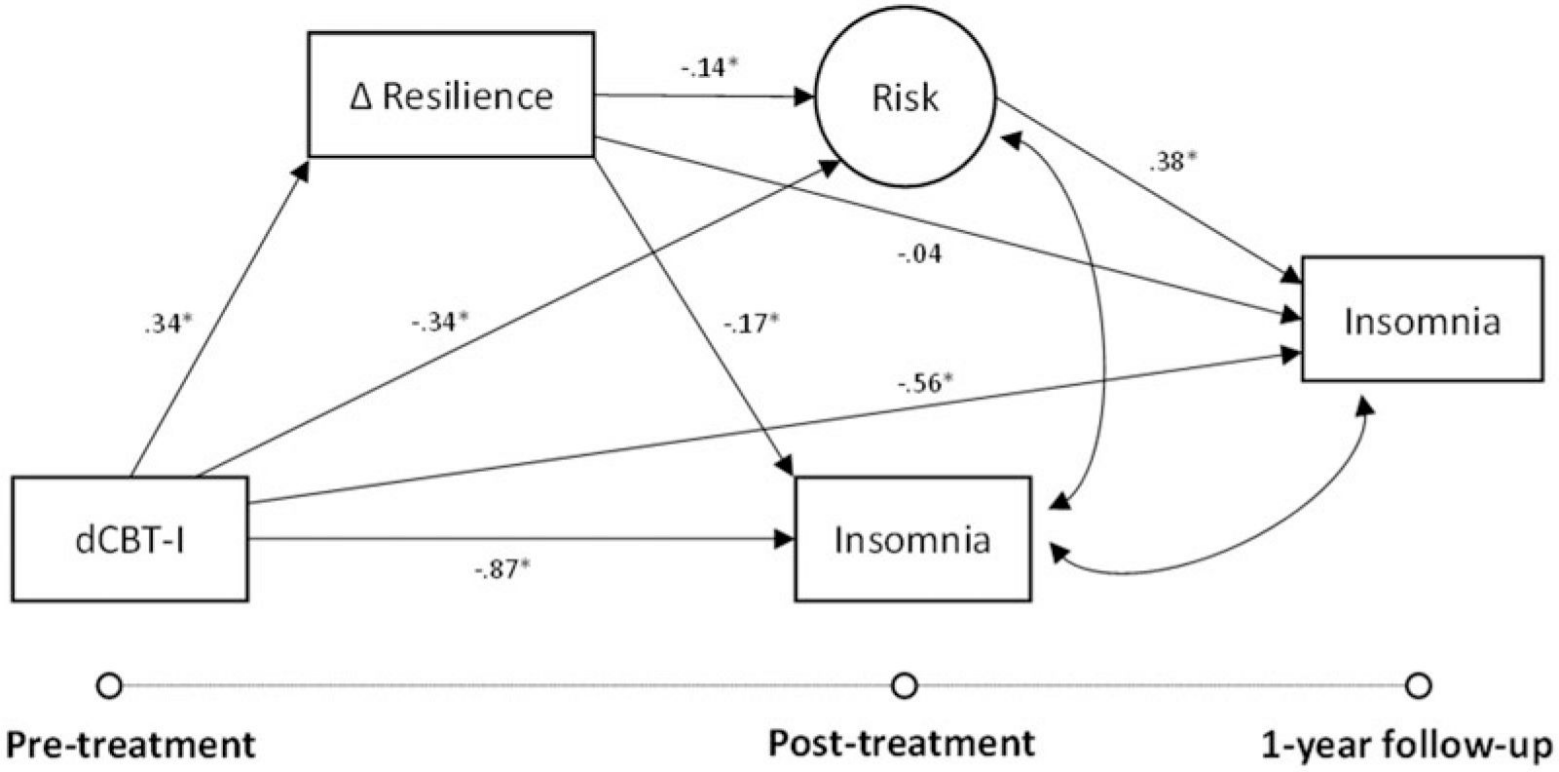
Structural equation model relating the impact of digital cognitive behavioral therapy for insomnia (dCBT-I) on insomnia severity at post-treatment (6–12 weeks after pre-treatment) and 1-year follow-up through changes in resilience and latent risk. Fit indices showed a good model fit to the data [χ^2^ (8, *N* = 658) = 13.8, CFI = 0.99; TLI = 0.99; RMSEA = 0.03].

The structural model (see Fig. 4) fit indices showed good model fit to the data [χ^2^ (8, *N* = 658) = 13.8, CFI = 0.99; TLI = 0.99; RMSEA = 0.03]. Consistent with the first hypothesis, results revealed that those in the dCBT-I condition exhibited greater improvements in resilience [*b* = 0.23 (*β* = 0.34), 95% CI 0.13–0.33]. Interestingly, improvement in resilience was also a significant mediator of the post-treatment gains in insomnia severity associated with dCBT-I [*b* = −0.33 (*β* = 0.06), 95% CI −0.54 to −0.17]. dCBT-I also reduced latent transdiagnostic risk at post-treatment compared to the control condition, which was also mediated by improved resilience [*b* = −0.33 (*β* = −0.05), 95% CI −0.57 to −0.16]. Finally, the indirect effect of reduced risk through improved resilience further predicted lower insomnia severity at 1-year follow-up [*b* = −0.12 (*β* = −0.02), 95% CI −0.24 to −0.05]. An additional sensitivity analysis in a subsample that achieved post-treatment remission indicated that each point improvement in resilience following treatment reduced the odds of insomnia relapse by 76% 1 year later (OR 0.24, 95% CI 0.12–0.45).

### Improved resilience as a mediator of treatment gains in depression

**Fig. 5.**
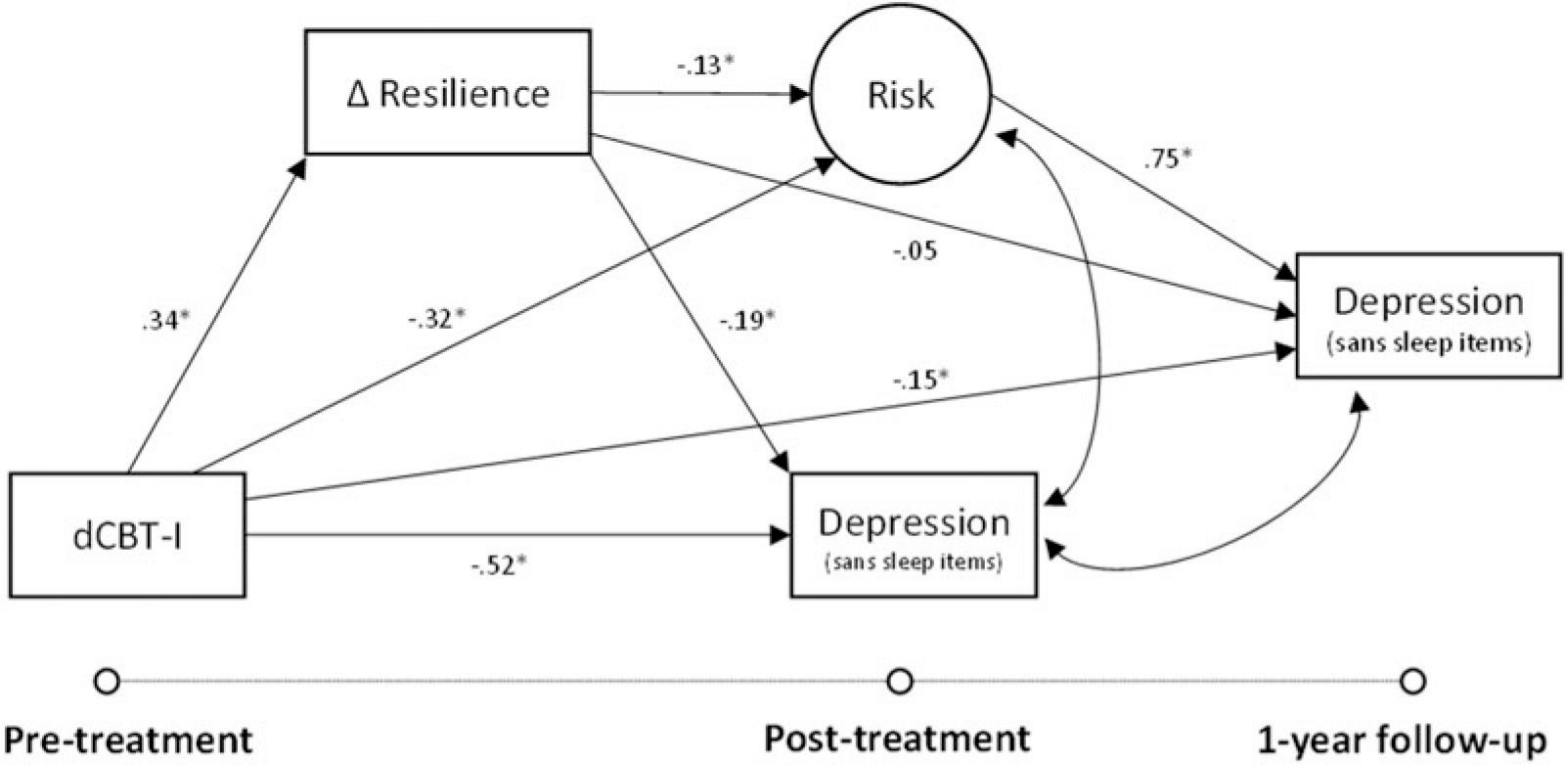
Structural equation model relating the impact of digital cognitive behavioral therapy for insomnia (dCBT-I) on depression severity (sans sleep items) at post-treatment (6–12 weeks after pre-treatment) and 1-year follow-up through changes in resilience and latent risk. Fit indices showed good model fit to the data [χ^2^ (8, *N* = 658) = 12.1, CFI = 1.00; TLI = 0.99; RMSEA = 0.03].

The structural model (see Fig. 5) fit indices for the depression model also indicated a good model fit to the data [χ^2^ (8, *N* = 658) = 13.5, CFI = 1.00; TLI = 0.99; RMSEA = 0.03]. Like the analysis with insomnia, the model with depression as the outcome variable of interest also revealed that greater improvement in resilience (i.e. from pre- to post-treatment) predicted lower post-treatment depression severity (sans sleep items) [*b* = −1.22 (*β* = −0.19), 95% CI −1.73 to −0.78]. Analyses also revealed that dCBT-I had an indirect effect on post-treatment depression severity through improved resilience [*b* = −0.28 (*β* = −0.06), 95% CI −0.47 to −0.14]. As found in the prior model, dCBT-I reduced latent transdiagnostic risk compared to the sleep education control [*b* = −2.19 (*β* = −0.32), 95% CI −3.00 to −1.39], and that reduced risk was also mediated by improved resilience [*b* = −0.31 (*β* = −0.05), 95% CI −0.55 to −0.14]. Finally, the indirect effect of reduced risk through improved resilience further predicted lower depression severity at 1-year follow-up [*b* = −0.15 (*β* = −0.03), 95% CI −0.28 to −0.07]. A sensitivity analysis in a subsample with minimal to no depression (full score) at baseline indicated that each point improvement in resilience following treatment reduced the odds of moderate to severe depression (full score) by 65% 1 year later (OR 0.35, 95% CI 0.18–0.65).

## Discussion

The overall aim of this study was to examine if insomnia treatment improved psychological resilience and test improved resilience as a candidate mechanism in the protective effects of CBT-I. Results provided support for our hypotheses that (1) psychological resilience improved with dCBT-I, and that (2) resilience protected against insomnia and depression 1-year later by reducing transdiagnostic risk (as indicated by sleep reactivity, stress, and rumination).

Importantly, this study is among the first to take a strengths-based approach as opposed to a deficits-based approach in understanding the mechanisms underlying the clinical impact of insomnia treatment. There is a plethora of research demonstrating that insomnia – especially when left untreated – is a robust risk factor for medical morbidities. However, less research has focused on how mitigating insomnia can promote health and resilience. Indeed, emergent research has suggested that insomnia treatment not only effectively removes a disease-state but may also have an ‘upward spiral’ effect in protecting against other illnesses, perhaps through improving stress reactivity and regulation. For example, treatment of insomnia not only reduces comorbid depression symptoms but also prevents future incidence of depression and relapse (Batterham et al., 2017; Cheng et al., 2019; van’t Veer-Tazelaar et al., 2009). One mechanism may be reductions in rumination as a component that perpetuates stress (Cheng et al., 2020b). Additionally, research during the COVID-19 pandemic has found that those who received dCBT-I prior to the pandemic were protected from more adverse outcomes – including lower stress – compared to those who received sleep education, despite being comparably impacted by the pandemic (Cheng et al., 2020a). Our results extend this research to more directly indicate that mitigating insomnia symptom severity leads to improved resilience, which protects against future adverse stressors and health outcomes.

Consistent with other findings in the literature, our results also indicate that while reducing insomnia bolsters resilience, the opposite also appears true – that reductions in insomnia associated with dCBT-I are also mediated by improved resilience (i.e. a bidirectional relationship, albeit to a smaller degree). Although dCBT-I does not explicitly target resilience (hence the smaller effect), its components may still bolster response and recovery from stress independently from improved sleep. For example, when deployed effectively, cognitive strategies taught in CBT-I (e.g. cognitive reappraisal, paradoxical intention, etc.) may help patients recover from stress more effectively. Furthermore, reductions in dysfunctional beliefs about sleep may also reduce perseverative thinking about sleepless nights, further aiding stress management and recovery. The improvement in stress management and recovery results in improved resilience, which then contributes to the acute reduction of and protection from future insomnia and depression.

Results from this study support a framework for conceptualizing how improvement in psychological resilience following dCBT-I underlies improvements in risk for insomnia and depression (related to stress reactivity) up to 1-year following treatment. Results found that those in the dCBT-I group showed a 4.9-point reduction in sleep reactivity (3.1 points more than the control group), which was partially mediated through improved resilience. Sleep reactivity is a robust predisposing risk factor for insomnia that describes a vulnerability in the sleep system to perturbations due to a range of stimuli including external/environmental (e.g. first night effect) (Chen et al., 2015; Kalmbach et al., 2018), psychological (Drake et al., 2011), and physiological (e.g. caffeine) (Drake et al., 2006). Our prospective research has shown that the development of insomnia disorder involves a sensitization of sleep reactivity (an average of 3.8 points) that increases the likelihood of symptom exacerbation or recurrence from stress (Kalmbach, Pillai, Arnedt, Anderson, & Drake, 2016a). As the reduction in sleep reactivity following dCBT-I is comparable in magnitude, these findings suggest that dCBT-I may reverse this sensitization via improved resilience, thereby mitigating sleep reactivity as a contributor to recurrent insomnia.

These results also suggest that dCBT-I may reduce stress as a risk factor of insomnia and depression. Indeed, not only did we see reduced stress following dCBT-I at 1-year follow-up, a follow-up to this study revealed that the improvements in stress persisted 3–4 years following treatment (during the COVID-19) (Cheng et al., 2020a), suggesting that the protective effects of dCBT-I are highly durable. In conjunction with prior research, it may be that resilience buffers against a more severe stress response via appraisals of stressors as less negative or unmanageable (Morin et al., 2003; Pillai et al., 2014; Voss et al., 2006). These results are also consistent with the literature on the importance of sleep in mood and emotion regulation, which is a critical component in stress recovery and thus resilience. Studies have shown that poor sleep is associated with heightened negative affect and blunted positive affect (Ong, Cardé, Gross, & Manber, 2011). Additionally, disturbed sleep also sensitizes attention to negative emotional stimuli and attenuates responsivity to positive events (Jackson et al., 2013; Zohar, Tzischinsky, Epstein, & Lavie, 2005). Under the stress-diathesis model, this increases risk for psychopathology as it not only enhances baseline stress, but also potentiates the stress response to negative stimuli and detracts from any protective impact of positive events. Conversely, restoring sleep health appears to attenuate risk as indicated by stress, sleep reactivity, and rumination, thus promoting more effective recovery from stressors. Indeed, results indicated that improvements in resilience contributed to the prevention of depression even after accounting for improvements in rumination as another established mechanism for depression prevention (Cheng et al., 2020b).

Some limitations should be considered in interpreting these results. First, psychological resilience relied on a self-report measure, albeit one that has been validated. Further research should replicate these findings with objective measures of psychological resilience. Second, individuals with severe depression with acute suicidal ideation were excluded from this study due to the aims of depression prevention; however, individuals did develop moderate to severe depression at the follow-up assessments. Finally, in alignment with the analytical approach, interpretation of parameter estimates should only be generalized to those with some exposure to dCBT-I.

## Conclusion

This randomized controlled trial examined stress and resilience following dCBT-I. Results demonstrated that dCBT-I improved psychological resilience, and that this improvement was a contributing mechanism to reducing risk for insomnia and depression 1 year later. These results suggest that, in addition to mitigating the disorder, insomnia treatment may also bolster protective factors that enhance adaptation and response to stressful life events. This has implications for insomnia treatment as a candidate strategy for stress inoculation in those at risk for adverse health outcomes.

## Figures and Tables

**Table 1.** Sample characteristics by treatment condition (mean ± s.d.; or %)

	Control (*n* = 300)	dCBT-I (*n* = 358)
Age	45.7 ± 15.8	44.5 ± 15.1
Sex (%F)	80.0%	77.9%
Insomnia severity (ISI)	17.7 ± 4.3	17.9 ± 4.3
Depression severity (QIDS-SR_16_ total score)	10.8 ± 4.6	10.8 ± 4.5
Resilience (BRS)	3.3 ± 0.9	3.2 ± 0.8
Stress	2.4 ± 1.0	2.4± 0.9
Sleep reactivity (FIRST)	24.6 ± 6.8	25.6 ± 6.4
Rumination (PTQ)	28.1 ± 13.70	26.9 ± 12.8
Race		
White	75.1%	67.0%
Black	18.2%	25.0%
Other	3.7%	8.0%
Education		
High school or less	14.5%	14.7%
Some college	26.3%	33.7%
College	38.8%	29.3%
Graduate school	20.4%	22.3%
Household income		
Low (<35k)	40.8%	44.4%
Middle (<75k)	29.2%	28.3%
Higher (75k+)	30.0%	27.3%

dCBT-I, digital cognitive behavioral therapy for insomnia; ISI, Insomnia Severity Index; QIDS-SR_16_, Quick Inventory of Depressive Symptomatology – 16-item self-report; BRS, Brief Resilience Scale; FIRST, Ford Insomnia Response to Stress Test.

No significant differences by condition were found.

## Footnotes

1 Those with and without exposure to treatment had near zero difference in resilience (*b* = 0.008, S.E. = 0.124, *p* = 0.952).

† The notes appear after the main text.
